# Progress in polydopamine-based melanin mimetic materials for structural color generation

**DOI:** 10.1080/14686996.2020.1852057

**Published:** 2021-01-22

**Authors:** Michinari Kohri

**Affiliations:** Department of Applied Chemistry and Biotechnology, Graduate School of Engineering, Chiba University, Chiba, Japan

**Keywords:** Structural color, melanin, polydopamine, polymer particles, biomimetic, 101 Self-assembly/Self-organized materials, 204 Optics/Optical applications, 212 Surface and interfaces

## Abstract

Structural color is a color derived from optical interaction between light and a microstructure and is often seen in nature. Natural melanin plays an important role in bright structural coloration. For example, the vivid colors of peacock feathers are due to structural colors. The periodic arrangement of melanin granules inside the feathers leads to light interference, and the black granules absorb scattered light well, resulting in bright structural color. In recent years, polydopamine (PDA) has attracted attention as a melanin mimetic material. This review article summarizes recent research on structural coloration using PDA-based artificial melanin materials. It also outlines possible applications using bright structural colors realized by artificial melanin materials and future perspectives.

## Graphical abstract

**Figure:**
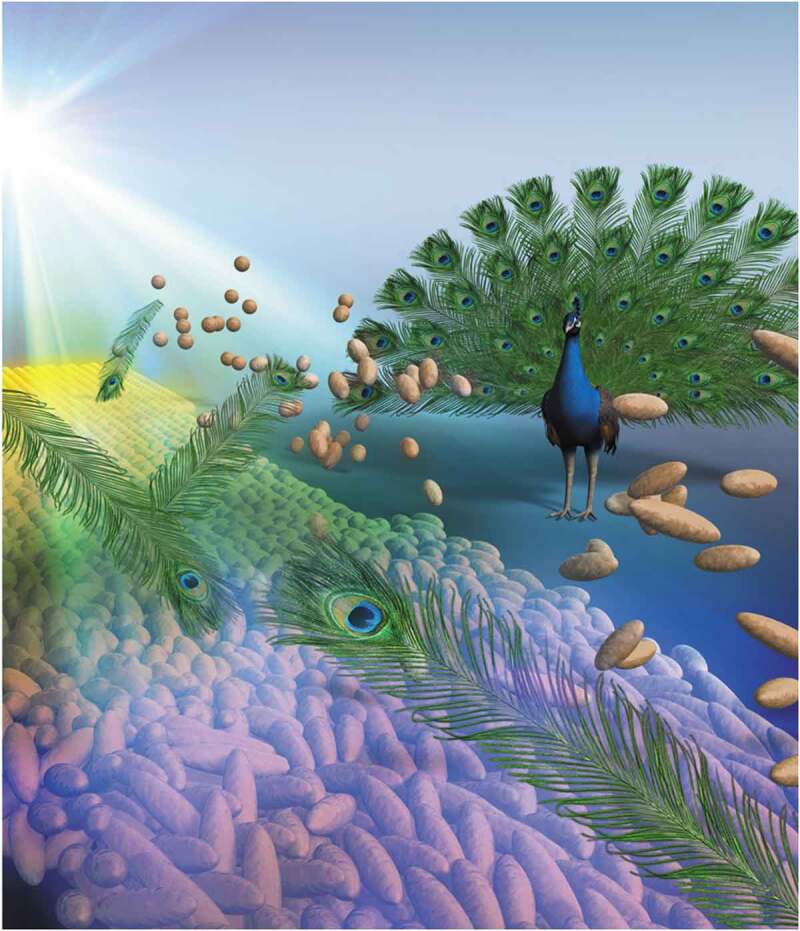

## 1. Introduction

**Figure 1.**
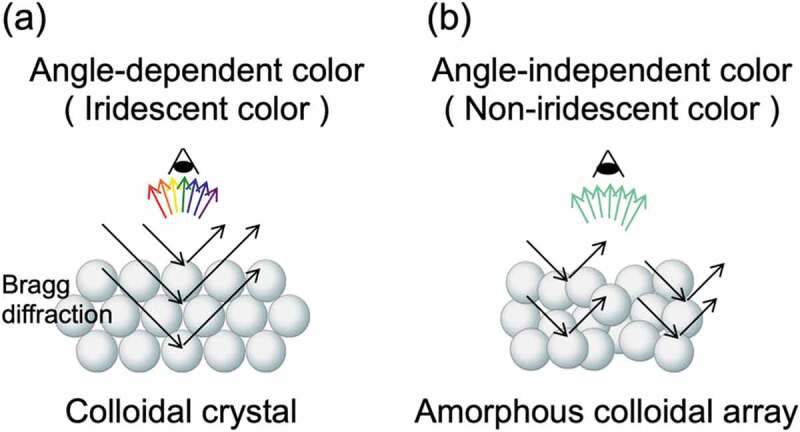
Two types of structure colors obtained by the assembly of colloidal particles: (a) colloidal crystals and (b) colloidal amorphous arrays

The structural color produced by the interaction of light and a microstructure does not fade as long as the microstructure is maintained, and light energy can be converted to color. Artificial fabrication of structural color materials is achieved by incorporating optical phenomena that generate structural colors, e.g. thin film interference, multilayer interference, diffraction, and scattering, into the material design to form a microstructure. The development of structural color materials that take advantage of the characteristics of each optical phenomenon is in progress [1–5]. In particular, colloidal crystals generated by assembling colloidal particles have a wide range of designs, and many studies on these materials have been conducted [6–10]. A structure in which monodisperse colloidal particles with a particle diameter of several hundred nm, close to the wavelength of light, are regularly arranged is called a colloidal crystal structure. The structural color produced by fcc (111) planes of the colloidal crystal structure is known to follow the Bragg-Snell Equation (1) shown below [11].

(1) $$m\lambda =\sqrt{\frac{8}{3}d^{2}\left(n^{2}-\sin^{2}\theta \right)}$$

where *m* is the diffraction order, *λ* is the wavelength of light, *d* is the interparticle distance, *n* is the effective refractive index of the colloidal crystal (particle and medium), and *θ* is the auxiliary angle of the incident light. Colloidal crystals produce highly reflective, angle-dependent structural colors due to the effects of Bragg diffraction (Figure 1(a)). In contrast, recent research has shown that when an amorphous structure with an intentionally disturbed arrangement structure is constructed, the scattering due to the particle size of the colloidal particles selectively enhanced, and angle-independent structural color can be obtained (Figure 1(b)) [12]. In the development of structural color materials, it is important to control the angle dependence of structural colors according to the application.

For structural colors produced by colloidal assemblies, the hue, saturation, and angle dependence of the color are controlled by parameters such as size, refractive index, blackness, and assembled structure of the particles used. Based on these parameters, the development of structural color materials is advancing from various perspectives [13]. In designing colloidal assemblies, in addition to the method of creating the microstructures, the materials that make them up are important. Research on structural color materials from the viewpoint of biomimetics is being actively conducted [14–17]. Another important challenge is the development of a method to generate highly visible structural colors from the assembly of colloidal particles. When light hits a submicron-sized microstructure that produces structural color, it appears white to the human eye due to multiple scattering of the light [18]. One effective way to produce a bright structural color is to dope the assemblies of colloidal particles with black substances that absorb the extra scattered light. It has been reported that doping with black substances such as carbon black [18–21], graphene [22–24], iron oxide nanoparticles [25,26], and polypyrrole [27] was effective in creating bright structural colors.

Structural colors are also found in the coloring of many organisms in nature, and melanin plays an important role in the generation of bright structural colors in these organisms. Over the past five years, research on structural color generation using polydopamine (PDA), which is a melanin-like substance inspired by structural coloration in nature, has rapidly progressed. This review article introduces the importance of melanin in the chromogenic mechanism of typical organisms with vivid structural colors and a recent topic on the development of structural colors with PDA-based artificial melanin materials.

## 2. The role of melanin in vivid structural colors in nature

**Figure 2.**
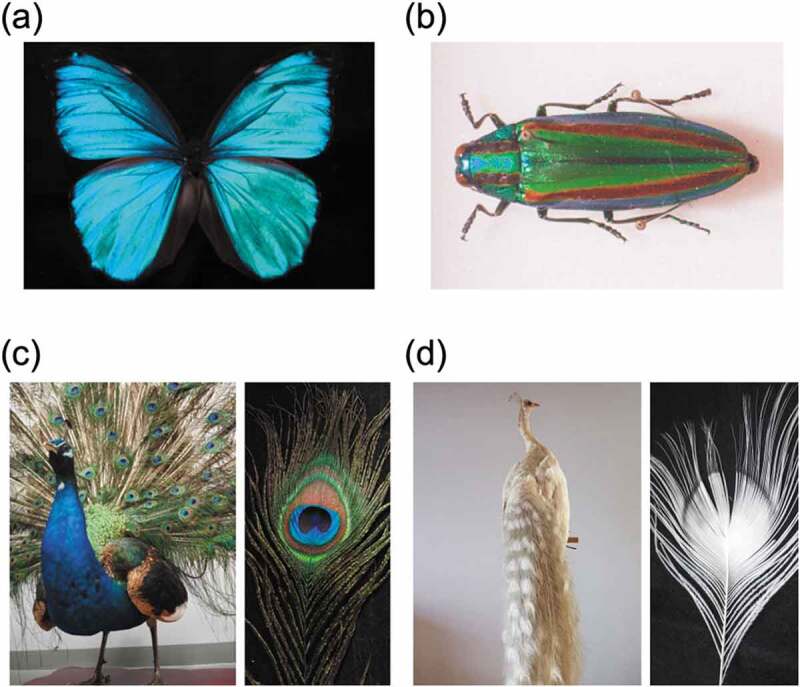
Photographs of (a) a *Morpho* butterfly, (b) a jewel beetle, (c) a peacock, and (d) an albino peacock. The stuffed peacocks shown in (c) and (d) are the property of the National Museum of Nature and Science

Structural colors are often found in the vivid coloring of natural organisms such as insects and birds. The beautiful blue scales of the *Morpho* butterfly are attributed to structural color due to the hierarchical shelf structure [28,29]. Melanin is a dark brown to black substance that protects the skin from ultraviolet rays and is well known as a component of human hair [30,31]. In addition to the periodicity of the microstructure, the presence of melanin is essential for the structural color of organisms that are characterized by bright colors. In general, when light is applied to a submicron-sized microstructure, the light is scattered and appears white to the human eye. Since melanin can absorb a wide range of visible light, if part or all of a microstructure is made of melanin, scattered light will be absorbed, and the structural color will be conspicuous and appear bright to the human eye (Figure 2(a)). On the *Morpho* butterfly scale, the melanin layer is underneath the shelf structure, the scattered light is properly absorbed, and a bright blue structural color can be seen [28,29]. The shiny green color of the wings of jewel beetles, also known as tiger beetles, is also a typical structural color derived from multilayer interference (Figure 2(b)). The jewel beetle wing is composed of a multilayer film in which approximately 20 layers of melanin and cuticle are alternately stacked, and the structural color is clearly visible because the melanin layer in the microstructure properly absorbs scattered light [32,33]. The refractive index of melanin is also important in structural coloration. Because the melanin layer has a high refractive index, the reflectance enhanced at the interface between the melanin layer and the cuticle layer with a low refractive index [34,35]. Yabu and coworkers reported highly reflective structural color films, consisting of alternating multilayers of transparent poly(1,2-butadiene) (PB) and the black metal osmium (Os), which mimic the multilayered structure of jewel beetles [36]. Since the refractive index of Os is much higher than that of PB, light was reflected at the interface between the two materials, causing strong reflection between multiple layers.

A famous example of structural coloration due to the microstructure formed by melanin itself is the color of peacock feathers (Figure 2(c)). Inside peacock feathers, rod-shaped melanin granules form a periodic microstructure that absorbs scattered light and creates a bright structural color [37–39]. Organisms that cannot produce melanin in vivo are called albino. As shown in Figure 2(d), albino peacock feathers, which lack the melanin microstructure, appear white [40]. In natural vivid structural coloration, melanin plays an important role both in microstructure building and suppression of scattered light.

## 3. Characteristics of polydopamine as a melanin mimetic material

**Figure 3.**
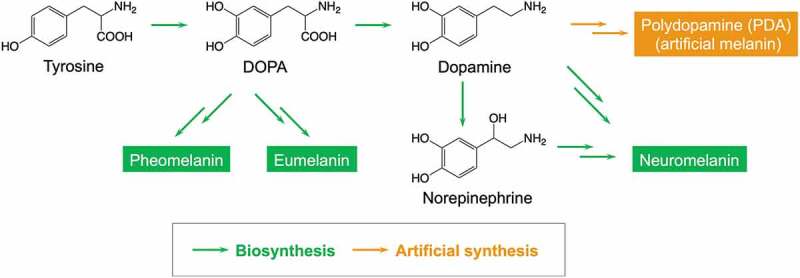
Biosynthesis of natural melanin and artificial synthesis of melanin mimetic materials, i.e. PDA

In vivo, melanin is produced from 3,4-dihydroxyphenylalanine (DOPA), an amino acid, and is classified into pheomelanin, eumelanin and neuromelanin depending on the composition (Figure 3) [30,31]. The complexity of the multistep enzymatic reaction makes it difficult to artificially synthesize microstructure-controlled melanin using the biosynthetic pathway. In 2007, Lee and coworkers reported that dopamine, a derivative of DOPA, was easily polymerized through self-oxidative polymerization to form PDA [41]. Due to the excellent adhesion of PDA to the surfaces of various substrates, research into the use of PDA as a surface modifier has developed rapidly. In addition to surface coating methods that utilize the adhesiveness of PDA, advanced materials such as PDA-based gel materials, conductive materials, catalytic materials, self-healing materials, and biomaterials have been developed. Although this review does not include the history and applications of PDA, some recent review articles provide more details in these areas [42–47].

Since dopamine is a metabolite of DOPA in vivo, from a chemical structure perspective, PDA has almost the same composition as natural melanin and can be considered artificial melanin [48]. From this viewpoint, research focusing on the structure-function relationship between natural melanin and PDA is progressing [49]. Inspired by the functionality of natural melanin, PDA-based functional materials such as free radical scavengers [50], UV absorbers [51], metal ion probes [52], catalyst [53,54], and antioxidants [55] have been developed.

## 4. Polydopamine particle-based structural coloration

**Figure 4.**
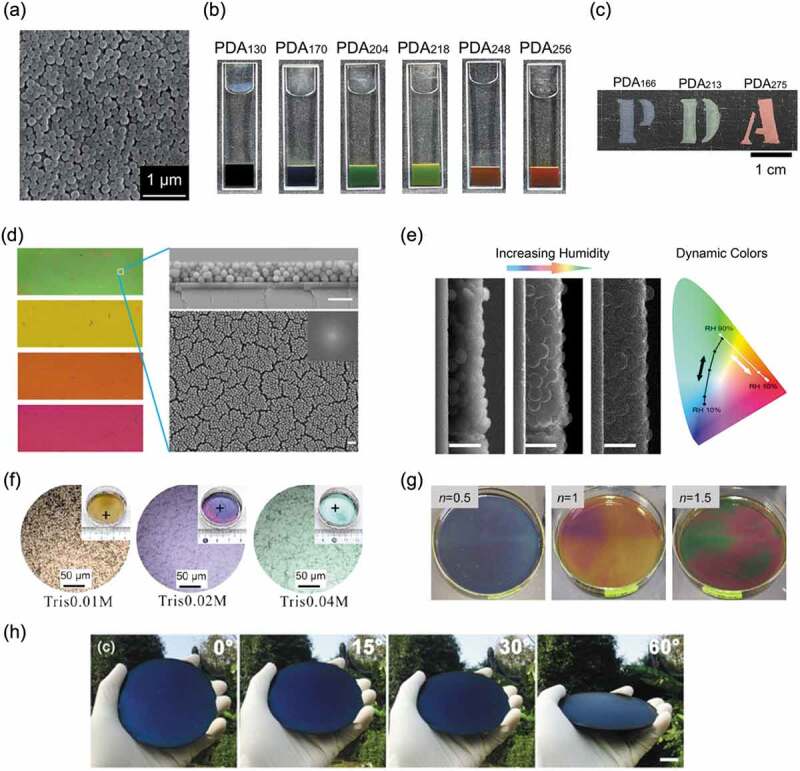
(a) SEM images of monodisperse PDA particles. (b) Scattering-derived structural colors from the assembly of submicron-sized PDA particles. (c) Structural colors achieved by spray coating of PDA particles. Reproduced with permission from [60] (Copyright 2015, Royal Society of Chemistry). (d) Structural coloration based on thin-film interference using PDA particles. Reproduced with permission from [64] (Copyright 2015, American Chemical Society). (e) Changes in structural color due to humidity. Reproduced with permission from [65] (Copyright 2016, American Chemical Society). (f) Structural color achieved by placing a thin PDA film on top of a layer of PDA particles. Reproduced with permission from [67] (Copyright 2015, American Chemical Society). (g) Structural coloration due to the hierarchical structure of the PDA thin film and PDA particles. Reproduced with permission from [68] (Copyright 2017, Society of Polymer Science). (h) Non-iridescent structural color of a PDA coating on a silicon wafer. Reproduced with permission from [71] (Copyright 2017, Royal Society of Chemistry)

Our group has conducted research on colorless functionalized PDA thin films [56–58] and morphology control of PDA thin films [59]. In the course of these studies, it was found that by polymerizing dopamine in a water/methanol mixed solvent, monodisperse PDA particles can be obtained. In 2015, our group reported that a bright structural color was observed from the assembled structure obtained by concentrating an aqueous dispersion of monodisperse PDA particles that mimic melanin granules (Figure 4(a,b)) [60]. These colors were scattering-derived structural colors that result from the assembly of submicron-sized PDA particles, and the color tone was controlled by simply selecting the size of particles used. In addition, the color tone could be changed by attaching hairy polymer chains to the surface of PDA particles. When PDA particles grafted with a hydrophilic hair polymer were assembled, the tone of the structural color was controlled by the change in interparticle distance [61]. Since PDA particles were well dispersed in water [62], structural coloration by spray painting was also possible (Figure 4(c)) [60]. By coating PDA particles, which were well dispersed in an aqueous solvent, with a magnetic surfactant, the dispersibility in organic solvents was improved, and the color was changed by a magnetic field [63]. The coloring mechanism in nature was reproduced, and bright structural coloration that suppressed light scattering was realized by constructing a microstructure with PDA particles of uniform size.

Structural coloration based on thin film interference using PDA particles has also been reported. Xiao and coworkers reported that PDA particles with a size of approximately 184 nm were assembled to form a thin film, and the tone of the structural color changed depending on the film thickness (Figure 4(d)) [64]. In this case, it is possible to control the color tone by changing the film thickness by utilizing the hygroscopicity of PDA particles (Figure 4(e)) [65]. A high refractive index of 1.74 for PDA particles has been shown to be important for vivid structural coloration. Eom and coworkers reported that structural color was observed from a film in which natural melanin particles obtained from squid ink were deposited by the layer-by-layer (LBL) method [66]. By controlling the film thickness with the LBL cycles, a wide range of iridescent colors were obtained. Structural coloration by thin film interference is possible, even if a PDA thin film prepared by directly polymerizing dopamine is used instead of a thin film in which PDA particles are assembled. Wu and coworkers obtained structural color by placing a thin PDA film on top of a layer of PDA particles that acted as a light absorbing layer (Figure 4(f)) [67]. Our group also demonstrated that when the polymerization of a dopamine-containing silane coupling agent was carried out under static conditions, structural color derived from the PDA thin film formed at the gas-liquid interface can be obtained (Figure 4(g)) [68]. At this time, the visibility of the structural color was enhanced by the PDA particles that were simultaneously generated on the liquid phase. Structural coloration due to the hierarchical structure of the PDA thin film and PDA particles can be regarded as a system that mimics the coloration of rock pigeon feathers [69,70]. Zang and coworkers reported that only coating a uniform PDA thin film on a silicon wafer yielded an angle-independent structural color (Figure 4(h)) [71]. They showed that large-scale and stimulus-responsive structural color films were synthesized by the co-deposition of PDA with poly(*N*-isopropylacrylamide). Vega and coworkers demonstrated structural color drawing by coating the surface of a silicon-silicon nitride wafer with a PDA film whose thickness varied depending on the patterned area [72].

## 5. High-visibility structural coloration by core-shell-type artificial melanin particles

### 5.1. Particle design

**Figure 5.**
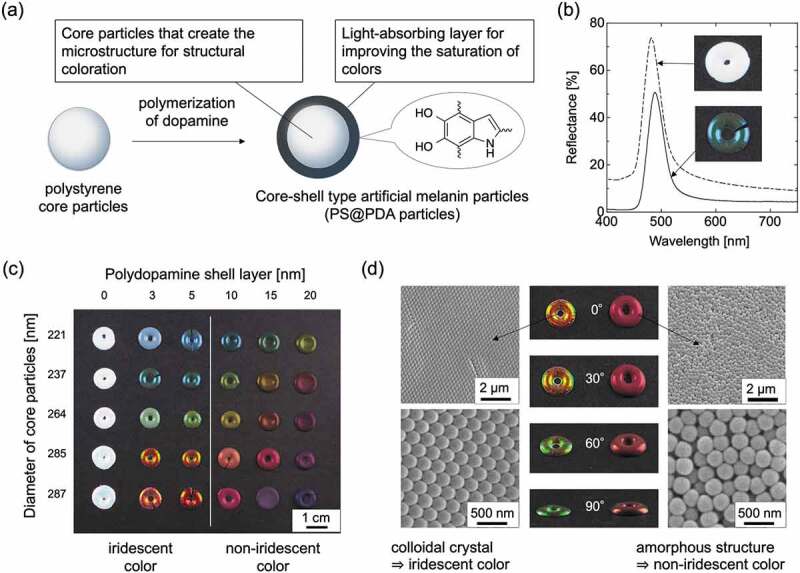
(a) Design of core-shell type artificial melanin particles, i.e. PS@PDA particles. (b) Reflection spectra of pellet samples from PS particles (dotted line) and PS@PDA particles (solid line). The insets show photographs of the pellet samples. (c) Structural color pellets prepared from PS@PDA particles with different core diameters and PDA shell thicknesses. (d) Photographs and SEM images taken from different view angles of structural color pellets composed of PS@PDA particles. Reproduced with permission from [73] (Copyright 2016, Nature Publishing Group)

Due to the extensive blackness of PDA particles, the structural color obtained by assembling PDA particles appears dark to the human eye. Thus, controlling the blackness of the particles is important for improving visibility. Our group reported high-visibility structural coloration by core-shell-type artificial melanin particles that can adjust the blackness, that is, the light absorption ability [73]. Core-shell-type artificial melanin particles were designed using monodisperse polystyrene (PS) particles as the core material and PDA as the shell layer. The synthesized particles were named PS@PDA particles. By changing the feed concentration of the dopamine monomer, a PDA shell layer of any thickness can be provided, and the blackness of the particles can be easily controlled. The monodisperse core particles are important for building the microstructure, and the melanin-mimetic PDA shell layer is important for absorbing scattered light (Figure 5(a)).

### 5.2. Highly-visibility structural color

Polymer particles such as PS particles are often used to form colloidal crystals. When the aqueous dispersion of PS particles was dropped on a silicone rubber plate, solid pellets were prepared by natural drying, and white pellets with an opal-like color were obtained. On the other hand, from the PS@PDA particles, highly visible structural color pellets were obtained. Figure 5(b) shows the results of the absolute reflectance measurement of the pellet surface, which was prepared from PS and PS@PDA particles. Although the reflectance of the peak at approximately 480 nm derived from blue structural color was higher for the pellets made of PS particles, the background due to light scattering was higher throughout the visible light range and appeared white to the human eye (dotted line in Figure 5(b)). On the other hand, the background of pellets of PS@PDA particles was reduced overall (solid line in Figure 5(b)). While the reflectance due to structural colors was also slightly reduced, the visibility to the human eye was dramatically improved, and highly visible structural coloration under normal ambient light was realized. This is because the light absorption layer composed of PDA appropriately absorbed the scattered light. By controlling the diameter of the core particles and the thickness of the PDA shell, it was possible to obtain nearly the full range of structural colors (Figure 5(c)) [73].

For practical use, the development of technologies for curing the colloidal crystal structure is important. Yi and coworkers reported the preparation of photonic crystals by co-assembly of PS@PDA particles and 3-aminopropyltriethoxysilane *via* a thermal-assisted self-assembly method [74,75]. Curing *via* a Schiff base reaction or Michael addition between the PDA shell layer and the silane coupling agent counteracts the tensile stress that is induced by latex shrinkage during drying, resulting in a crack-free photonic crystal that is resistant to wetting and impact. Chen and coworkers showed that co-assembly of polyurethane dispersions and PS@PDA particles resulted in structural color films with controlled angular dependence due to strong Brownian motion under heating conditions [76]. Liu and coworkers reported that alkaline vapor treatment improved the adhesion and robustness of amorphous colloidal arrays prepared by spray coating PDA-coated silica (SiO_2_) particles, i.e. SiO_2_@PDA particles [77]. This is because ammonia vapor provided a moist alkaline environment, which promoted further oxidative self-polymerization between PDA shells and cured samples. Shen and coworkers reported that the presence of chitosan during the natural drying of an aqueous dispersion of PS@PDA particles significantly suppressed the occurrence of cracks and improved the mechanical stability of the structural color coating [78]. They showed that structural color coating was possible not only on rigid substrates but also on flexible cotton fabrics that offer robust mechanical stability.

### 5.3. Effect of assembled structure on color

There are two types of structural colors, i.e. iridescent and non-iridescent structural colors, depending on whether the color is angle dependent or not. The color of a peacock feather is iridescent structural color arising from the regularly assembled structure of rod-shaped melanin granules, and the hue of the structural color changes depending on the viewing angle (*vide supra*) [37–39]. On the other hand, inside the feathers of *Cotinga maynana*, an amorphous structure arises from the keratin-based spongy matrix and air, and non-iridescent structural color is observed [79]. This is because the amorphous structure does not have long-range order, maintains short-range order, and causes selective reflection according to the lattice size. Controlling the angle dependence is an important issue in the use of structural colors [80,81]. Interestingly, structural color pellets prepared by assembling PS@PDA particles can easily control the angular dependence of the structural color depending on the thickness of the PDA shell layer [73]. Particles with a thin shell layer (< 5 nm) formed a close-packed colloidal crystal structure, producing an iridescent structural color (Figure 5(d) left). On the other hand, particles with a thick shell layer (> 10 nm) showed a non-iridescent structural color due to the disordered amorphous structure (Figure 5(d) right). When the PDA layer was thicker, the particle surface became rougher, which disturbed the particle arrangement and suppressed the formation of the close-packed structure. The great advantage of this method is that it is easy to control the assembled structure of the particles and the angular dependence of the structural color simply by controlling the PDA layer thickness.

**Figure 6.**
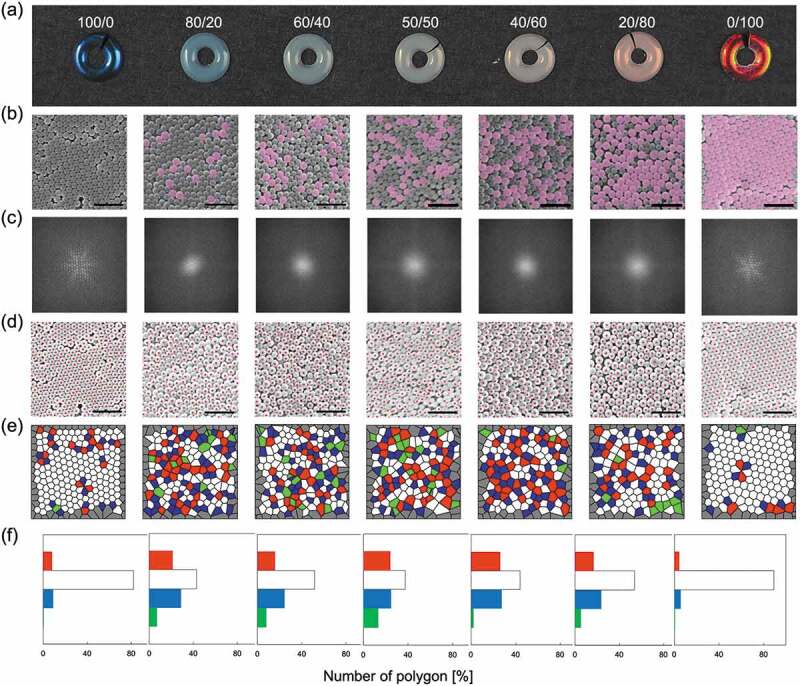
(a) Photographs and (b) SEM images of pellets obtained by mixing PS@PDA particles with different particle sizes. The scale bars are 1 μm. Large particles in the SEM images shown in (b) are marked in pink. (c) Fourier transform spectra and (d) determination of the particle centers of the SEM images shown in (b). (e) Voronoi diagram and (f) number of polygons. Red: 5-sided polygons, white: hexagonal polygons, blue: 7-sided polygons, green: others. Reproduced with permission from [82] (Copyright 2017, American Chemical Society)

It is also possible to control the angular dependence of the structural color by mixing particles [82]. When PS@PDA particles with different particle diameters were mixed to form a pellet, the particle arrangement changed depending on the mixing ratio, and the presence or absence of angle dependence was controlled. In this case, the average particle size was adjusted stepwise by the mixing ratio of the particles. As shown in Equation (1), the structural color depends on the particle size, so mixing the particles resulted in a neutral structural color (Figure 6(a)). The randomness of particle arrays is usually discussed in terms of Fourier transform images of scanning electron microscope (SEM) or transmission electron microscope (TEM) images [80,81]. Figure 6(b,c) show the SEM images of the pellet samples obtained by particle mixing and their Fourier transform images. On the surface of a pellet composed of one type of particle, the particles formed a close-packed structure, and a clear diffraction pattern was observed in the Fourier transform image. As the proportion of particle mixing increased, the particle arrangement became disordered, and a circular pattern was observed in the Fourier transform image. Although evaluation with a Fourier transform image is easy, it is difficult to visualize the degree of randomness of particle arrays. A Voronoi diagram is a diagram in which multiple points (seeds) at arbitrary positions in a metric space are divided into regions close to other points in the same metric space. Our group showed that the randomness of the array of particles can be visualized by a Voronoi diagram in SEM images using the center of the particle as a seed (Figure 6(d,e)) [82]. The randomness of a particle arrangement can be assessed by the proportion of hexagonal white polygons due to the close-packed structure (Figure 6(f)). The tone and angular dependence of the structural color can be adjusted simply by mixing particles, and it is expected to be applied to various coloring systems.

### 5.4. Effect of melanin precursor on color

While melanin is the black component of human hair, each person has a different hair color. This is due to the difference in melanin content and melanin composition. In the abovementioned system, an example using dopamine as a starting material for producing artificial melanin was shown. Recent studies have shown that in addition to dopamine, DOPA and norepinephrine, which occur before and after the in vivo metabolic pathway of dopamine, were also oxidatively polymerized to form polyDOPA and polynorepinephrine (PNE) [83–85]. From these findings, our group has shown that core-shell type artificial melanin particles were prepared by using DOPA and norepinephrine as starting materials [86]. Although an oxidizing agent such as sodium periodate was required for the progress of polymerization, core-shell particles were prepared even when DOPA and norepinephrine were used as monomers, and structural coloration with high visibility was possible. In particular, when norepinephrine was used, the smoothness of the particle surface after generation was higher than that when dopamine or DOPA was used, and as a result, a structural color material with high angle dependence was easily obtained. The differences in the intermediates formed during the oxidative polymerization of dopamine and norepinephrine affected the roughness of the resulting PDA and PNE thin films [87,88]. It was found that the properties of the artificial melanin particles produced change depending on the type of melanin precursor monomer, and the structural color that develops is also affected.

### 5.5. Effect of particle shape on color

**Figure 7.**
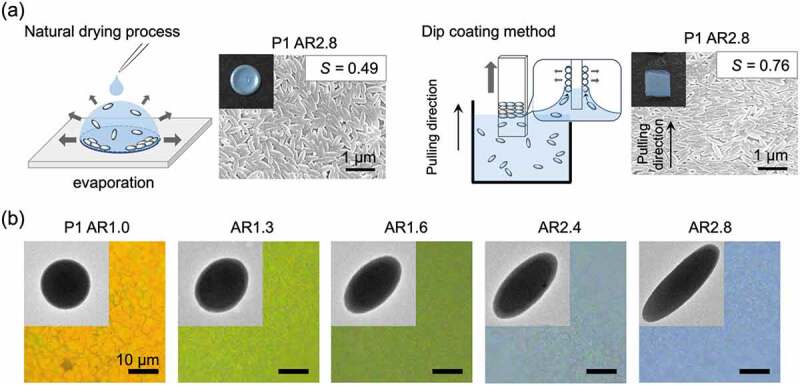
(a) SEM image of a sample in which ellipsoidal PS@PDA particles with an aspect ratio of 2.8 were assembled by a natural drying process and a dip coating method. The insets show photographs of the structural color samples. (b) TEM images of ellipsoidal PS@PDA particles with different aspect ratios and structural colors obtained by assembling those particles. Reproduced with permission from [89] (Copyright 2019, American Chemical Society)

In the preparation of artificial structural color materials in which colloidal particles are assembled, spherical and solid particles are generally used as building blocks. Looking at nature, the shapes of melanin granules, which are the constituents of structural coloration, are diverse. As mentioned above, in the structural color of peacock feathers, rod-shaped melanin granules are used as a building block [37–39]. Mimicking the shape of the building block, our group reported the structural coloration from ellipsoidal artificial melanin particles [89]. Ellipsoidal artificial melanin particles were obtained by stretching a polyvinyl alcohol (PVA) film containing PS@PDA particles at a temperature above the glass transition temperature of the core PS particles, cooling the sample, and then melting the PVA. The aspect ratio of particles was controlled from 1 to 2.8 depending on the degree of film stretching. When the ellipsoidal artificial melanin particles were dried on the substrate, they tended to deposit sideways on the substrate. This trend was more clearly observed when ellipsoidal artificial melanin particles were deposited on the substrate by dip coating instead of natural drying (Figure 7(a)). As a result, the minor axis length of the ellipsoidal particles affected the structural coloration, and the structural color blueshifted as the aspect ratio increased (Figure 7(b)).

Hollow-shaped melanin granules are also found as a material to build the microstructure to create the structural color of bird feathers [90,91]. Yi and coworkers reported that the hollow PDA amorphous colloidal structure exhibited an angle-independent structural color [92]. They obtained hollow PDA materials by curing the PS@PDA particle assembled structure with 3-aminopropyltriethoxysilane and then removing the PS core particles by solvent treatment. Our group demonstrated the preparation of an inverse opal structure, which was prepared by depositing PS@PDA particles on the substrate by the dip coating method, curing the sample with a photocuring resin, and then removing the PS site by solvent treatment [93]. In this case, a robust and bright structural color material with excellent angle dependency was obtained. A material with such a hollow PDA structure immobilized thereon is expected to have use as a sensor because the reflection wavelength changes depending on the refractive index of the solvent when immersed in the solvent. It is also possible to produce hollow melanin particles without curing the particle arrangement. Multiple coatings of the PDA shell layer resulted in artificial melanin particles with a thick and tough shell layer [94]. Our group showed that hollow artificial melanin particles were obtained by dissolving the core material by solvent treatment of core-shell particles having thick PDA or PNE shell layers [86]. As determined from Equation (1), the wavelength of the structural color is highly dependent on the refractive index of the material. Since the voids inside the hollow artificial melanin particles contained air, the refractive index differed from that of solid particles, and the structural color tone of the pellet samples changed significantly.

### 5.6. Effect of the PDA composite method on color

As mentioned above, the assembled structure of PDA particles or core-shell particles with a PDA shell layer was useful for producing bright structural colors. On the other hand, according to previous research, the visibility of the structural color can be improved by forming colloidal assemblies by doping black substances. Research is also progressing from the perspective of doping melanin materials. Zhang and coworkers showed that bright structural colors were observed by doping non-spherical natural melanin particles with an average size of approximately 110 nm, obtained from cuttlefish ink, when making an assembled structure composed of PS particles [95]. While there were limits to the size and uniformity of natural melanin particles, the visibility of the structural color was improved simply by doping the melanin material. Our group investigated the effect of the PDA composite method on structural color by comparing the binary co-assembly of cerium(IV) oxide (CeO_2_) particles and PDA particles of the same size with the unary assembly of CeO_2_@PDA core-shell particles [96]. In the binary system, the distorted shape of CeO_2_ particles disturbed the particle arrangement, resulting in a non-iridescent structural color. While color unevenness may occur, bright structural colors with the same tone were obtained by simply mixing PDA particles. On the other hand, the PDA coating produced uniform core-shell particles and formed a colloidal crystal structure that gave an iridescent structural color with high visibility. By controlling the thickness of the PDA shell, it was possible to change the color tone according to the particle size.

## 6. Potential applications

### 6.1. Ink application

**Figure 8.**
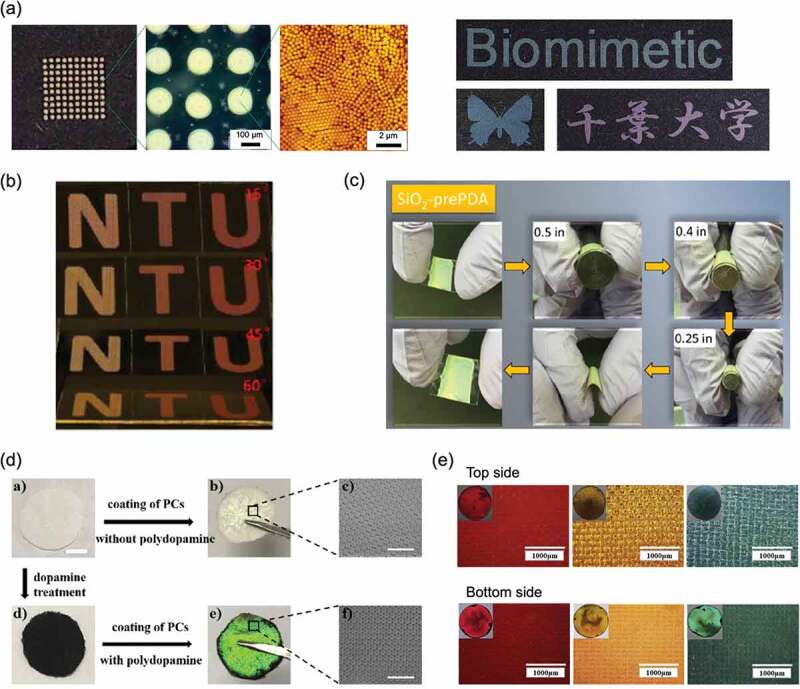
(a) Structural color printing by inkjet using an aqueous dispersion of PS@PDA particles. Reproduced with permission from [98] (Copyright 2019, Nature Publishing Group). (b) IFAST printing of PS@PDA particles. Reproduced with permission from [99] (Copyright 2019, American Chemical Society). (c) Structural color film made on a flexible PET substrate by EPD of pre-PDA and SiO_2_ particles. Reproduced with permission from [100] (Copyright 2019, American Chemical Society). (d) Structural colors obtained by assembling polymer particles on fabrics with and without PDA coating. Reproduced with permission from [102] (Copyright 2019, Elsevier). (e) Structural colors obtained by building a colloidal crystal structure consisting of PS@PDA particles on both sides of the fabric. Reproduced with permission from [103] (Copyright 2019, Elsevier)

Background color when observing structural colors is important for the development of ink materials based on structural colors. When observing the structural color on a white background such as paper, the visibility of the structural color is reduced due to the scattering of light. Therefore, a black background is usually used to improve the visibility of structural colors. Our group reported that by using PS@PDA particles that can absorb light, it was possible to observe bright structural colors not only on a black background but also on a white background [97]. The PS@PDA particles were well dispersed in an aqueous medium due to their high zeta potential (approximately −50 mV) [73]. Taking advantage of this feature, our group succeeded in structural color printing by the inkjet method using an aqueous dispersion of PS@PDA particles (Figure 8(a)) [98]. The sample after inkjet printing on the overhead projector film had a dome-shaped structure consisting of particle assemblies. The inkjet method has achieved the structural color printing of each color according to the particle size. Bai and coworkers reported the usability of infiltration-assisted (IFAST) printing of PS@PDA particles on porous substrates for large-scale structural color printing in addition to inkjet printing [99]. By IFAST printing in a nonequilibrium thermodynamic state with strong downward infiltration, the particles formed an amorphous colloidal array, providing an angle-independent structural color (Figure 8(b)).

Echeverri and coworkers demonstrated a large area structural color coating technique using electrophoretic deposition (EPD) [100]. By performing EPD of SiO_2_ particles in the presence of the precursor of PDA (pre-PDA), a structural color film was obtained on the flexible polyethylene terephthalate (PET) substrate. The pre-PDA enhanced the mechanical properties and enabled flexibility of the structural color coating with bending resistance, showing the possibility of application to flexible displays (Figure 8(c)). Dyeing using structural colors is also performed using the adhesive properties of PDA. Shi and coworkers reported the creation of a structurally colored fabric by coating cotton fabric with monodisperse polymer particles with carboxyl groups and pre-PDA [101]. The resulting fabric retained its original structural color even after 10 washes or heating to 100°C. They also showed that by assembling polymer particles on a PDA-coated fabric, the black background of the PDA thin layer significantly enhanced the color saturation of the structural color (Figure 8(d)) [102]. Wang and coworkers demonstrated that by building a colloidal crystal structure consisting of PS@PDA particles on both sides of the fabric, bright structural colors were obtained from the entire surface of the fabric (Figure 8(e)) [103].

### 6.2. Pigment application

A spherical photonic material with a three-dimensional structure constructed from colloidal particles is expected to have use as a structurally colored pigment, and many attempts have been made to achieve this application [104–108]. Our group demonstrated the fabrication of spherical photonic materials by the membrane emulsification method, utilizing the property that PS@PDA particles were well dispersed in water [109]. After making a monodisperse water-in-oil (W/O) emulsion containing PS@PDA particles and evaporating the solvent, a spherical photonic material was obtained, which showed structural color even when dispersed in the solvent. In addition to spherical photonic materials, a combination of microfluidic emulsification and solvent diffusion has also successfully created structural color fibers, which are fibrous photonic materials. Liu and coworkers also reported the microfluidic-assisted fabrication of spherical photonic materials using SiO_2_@PDA particles [110]. The resulting spherical photonic material exhibited structural color depending on the size of the SiO_2_@PDA particles. Additionally, biomolecules were immobilized on the surface of spherical photonic materials *via* the PDA layer, demonstrating their application in bioassays. Xiao and coworkers reported that spherical photonic materials were obtained by a one-pot reverse emulsion process using PDA@SiO_2_ core-shell particles, which have a melanin core with a high refractive index (~1.74) and a silica shell with a low refractive index (~1.45) [111]. They designed PDA@SiO_2_ particles by finite-difference time-domain simulations and reported that the refractive index of the material is important for structural coloration. These three-dimensional photonic materials may be used in various applications by adding them to existing materials.

### 6.3. Anti-counterfeiting application

**Figure 9.**
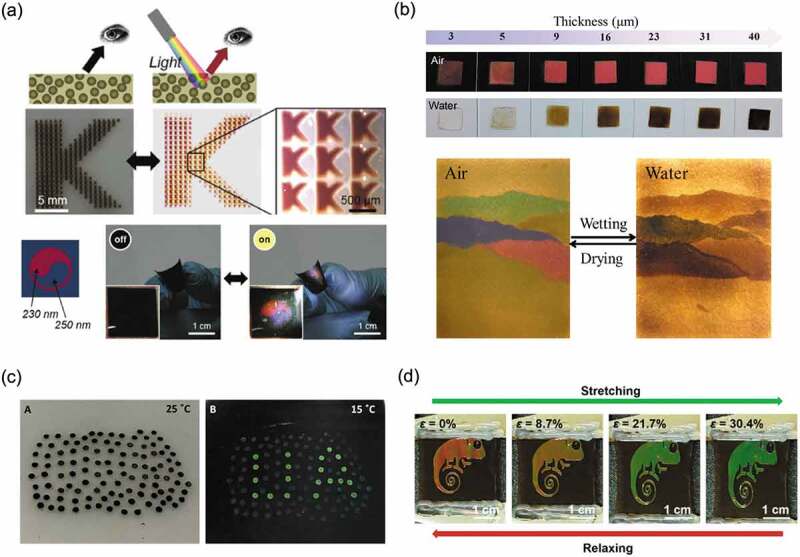
(a) Structural color observed from PDA particles in a solid matrix when illuminated by strong light. Reproduced with permission from [112] (Copyright 2017, Wiley-VCH). (b) Photographs of structurally colored arrays fabricated by SiO_2_@PDA particles exposed in air and water. Reproduced with permission from [113] (Copyright 2019, American Chemical Society). (c) Structural color pattern visible by decreasing the temperature of a binary assembly structure of SiO_2_ and PDA particles printed on a thermochromic epoxy resin. Reproduced with permission from [116] (Copyright 2020, American Chemical Society). (d) Reversible color changes due to stretching and relaxation of mechanochromic materials composed of elastomers containing binary colloidal arrays of SiO_2_ and PDA particles. Reproduced with permission from [121] (Copyright 2019, American Chemical Society)

The structural color obtained by assembling the PDA particles appears dark to the human eye due to the high blackness of the PDA particles. Therefore, to obtain structural color, it is necessary to darken the environment and illuminate the sample with a strong light source. Cho and coworkers reported an application that positively exploited the property that the hue of the structural color changed before and after exposing the assembled PDA particle structure to light [112]. As shown in Figure 9(a), when a sample that appeared black due to the light absorption of PDA was irradiated with strong light, the structural color was selectively observed. The PDA particles in the solid matrix showed structural color only when illuminated with strong light, and the construction of arbitrary patterns showed their utility as anti-counterfeit applications. Liu and coworkers reported an anti-counterfeiting system inspired by *Diphylleia grayi* whose color tone changes before and after getting wet with rain [113]. They showed that when the SiO_2_@PDA array was wetted with water, the original structural color disappeared, and the array appeared dark brown due to the absorption capacity of the PDA (Figure 9(b), upper row). The brown hue could be controlled by the SiO_2_@PDA particle array thickness since the dark brown color was dependent on the array thickness. Paintings created by varying the size of the SiO_2_@PDA particles and the thickness of the array showed different patterns before and after wetting (Figure 9(b), lower row). The background color is important for the visualization of structural colors, and research focusing on this phenomenon is progressing. For example, it has been reported that structural color was visualized upon changing from a white background to a black background using a polarizing plate [114,115]. Echeverri and coworkers demonstrated a system that visualizes structural color with temperature by creating a binary assembled structure of SiO_2_ and PDA particles on an epoxy resin film containing thermochromic microcapsules that change from white to black with temperature (Figure 9(c)) [116]. Additionally, it was shown that colloidal assemblies were efficiently constructed with a small amount of colloidal dispersion by using a substrate whose surface was perfluorinated by the cold plasma method. Our group also showed that the surface properties of the substrate were important in the formation of colloidal assemblies [117]. The formation of colloidal crystal structures of PS@PDA particles on substrates was shown to be controlled by creating a surface whose wettability and adhesion were controlled by external stimuli.

### 6.4. Sensing application

Structural color-based sensing readouts are directly visible to the human eye without the need for instrumental measurements. One useful technique is the formation of colloidal crystal structures on elastomers such as rubber, which change the structural color upon stretching [112–120]. Lee and coworkers reported the preparation of mechanochromic materials composed of elastomers containing binary colloidal arrays of SiO_2_ and PDA particles [121]. Reversible color changes due to stretching and relaxation were demonstrated. In this case, as the elastomer was created by photocuring, various designs of structural color patterns were created by photolithography techniques (Figure 9(d)). Sensing with improved visibility of structural colors was achieved because the composite PDA particles reduced incoherent scattering. These materials are expected to have use as sensors that can detect local strain and stress with high sensitivity.

## 7. Summary and perspective

Artificial melanin materials based on PDA were created by mimicking natural melanin, which is important for vivid structural coloration in nature [98,122,123]. By constructing a microstructure with black artificial melanin materials or incorporating them into the microstructure, the reflection and absorption of light were properly controlled, and highly visible structural coloration was realized. Since PDA is a highly biocompatible polymer that utilizes substances that are present in the living body, it may be useful to develop applications such as cosmetics that come into contact with the skin and safe and secure coloring agents, in addition to ink, anti-counterfeiting, and sensing materials. While the usefulness of artificial melanin-based structural color materials has been shown, the mechanism for constructing a periodic structure composed of natural melanin in vivo is still unknown. PDA is an easy-to-handle material that mimics natural melanin. In the future, it is expected that the knowledge introduced in this review article will contribute not only to practical research but also to the progress of basic research for understanding the development of structural colors in nature.
